# Severe Form of ßIV-Spectrin Deficiency With Mitochondrial Dysfunction and Cardiomyopathy—A Case Report

**DOI:** 10.3389/fneur.2021.643805

**Published:** 2021-04-27

**Authors:** Aziza Miriam Belkheir, Janine Reunert, Christiane Elpers, Lambert van den Heuvel, Richard Rodenburg, Anja Seelhöfer, Stephan Rust, Astrid Jeibmann, Michael Frosch, Thorsten Marquardt

**Affiliations:** ^1^Department of General Paediatrics, Metabolic Diseases, University Children's Hospital Muenster, Münster, Germany; ^2^Translational Metabolic Laboratory, Department of Paediatrics, Radboud Center for Mitochondrial Medicine, Radboud UMC, Nijmegen, Netherlands; ^3^Institute of Neuropathology, University Hospital Muenster, Münster, Germany; ^4^Department of Children's Pain Therapy and Paediatric Palliative Care, Faculty of Health—School of Medicine, Witten/Herdecke University, Witten, Germany

**Keywords:** ßIV-spectrin deficiency, mitochondrial dysfunction, neurodegeneration, cardiomyopathy, psychomotor developmental arrest, case report

## Abstract

ßIV-spectrin is a protein of the spectrin family which is involved in the organization of the cytoskeleton structure and is found in high quantity in the axon initial segment and the nodes of Ranvier. Together with ankyrin G, ßIV-spectrin is responsible for the clustering of KCNQ2/3-potassium channels and NaV-sodium channels. Loss or reduction of ßIV-spectrin causes a destabilization of the cytoskeleton and an impairment in the generation of the action potential, which leads to neuronal degeneration. Furthermore, ßIV-spectrin has been described to play an important role in the maintenance of the neuronal polarity and of the diffusion barrier. ßIV-spectrin is also located in the heart where it takes an important part in the structural organization of ion channels and has also been described to participate in cell signaling pathways through binding of transcription factors. We describe two patients with a severe form of ßIV-spectrin deficiency. Whole-exome sequencing revealed the homozygous stop mutation c.6016C>T (p.R2006^*^) in the SPTBN4 gene. The phenotype of these patients is characterized by profound psychomotor developmental arrest, respiratory insufficiency and deafness. Additionally one of the patients presents with cardiomyopathy, optical nerve atrophy, and mitochondrial dysfunction. This is the first report of a severe form of ßIV-spectrin deficiency with hypertrophic cardiomyopathy and mitochondrial dysfunction.

## Introduction

ßIV-spectrin is a protein of the spectrin-family, localized in the axon initial segment (AIS) and the nodes of Ranvier (1). Spectrins are proteins playing an important role in the organization and stabilization of neurons. They build tetramers of two α and two ß subunits. Five ß-spectrins ßI—ßV are known to be existent in human neurons (2).

In order to maintain the cytoskeletal structure, ßIV-spectrin forms periodical rings in defined intervals with αII-spectrin and actin, linked to ankyrin G (3). Spectrin is linked to the rings formed by actin and located every 180 nm around the axon (4). For stabilization, this complex is bound to ankyrin G by their N-terminal region (3). Binding of ßIV-spectrin to ankyrin G is important for clustering of KCNQ2/3 potassium- and NaV sodium channels in the cytoskeleton membrane. These channels are highly expressed in the AIS, where the action potential is generated and in the nodes of Ranvier, being important for the saltatory conduction (5).

Moreover, the actin cytoskeleton is described to build the anchorage for mitochondria (6).

Together with ankyrin G, ßIV-spectrin is essential for clustering of the cytoskeleton, for the preservation of the membrane potential and for the initiation of the action potential (7).

ßIV-spectrin helps to preserve the diffusion barrier of the axon through interaction with actin (8). A disturbance of this cytoskeletal diffusion boundary could lead to an impaired membrane diffusion of proteins (8).

ßIV-spectrin plays an important part in the organization of cardiac structure and likewise ßIV-spectrin was detected to contribute to cell signaling pathways in the heart (9).

In the heart ßIV-spectrin is localized in myocyte intercalated discs and has a major role in the organization of ion channels like cardiac voltage-gated sodium channels, Na_v_1.5 or potassium channels, like TREK-1 (10). ßIV-spectrin mutant mice have shown impairments in cardiac repolarization and arrhythmia (9). Another model with ßIV-spectrin mutant mice has shown impaired cardiac function through the activation of cardiac fibrosis. ßIV-spectrin binds the transcription factor STAT3, a transcriptional regulator influencing cell responses like hypertrophy and proliferation (11). Loss of ßIV-spectrin results in impaired binding of STAT3 leading to its activation. Consequently STAT3 accumulates in the nucleus and activates cardiac fibroblasts, leading to cardiac dysfunction (11).

Mutations in the SPTBN4 gene, coding for ßIV-spectrin, lead to defective clustering of the AIS and the Nodes of Ranvier, disturbed axonal membrane polarity, as well as the inability to generate action potentials inducing neurodegeneration (5, 12, 13). These mutations have been identified in seven individuals by Knierim et al. and Wang et al. causing congenital myopathy, neuropathy and central deafness (14, 15). So far no mitochondrial dysfunction or cardiomyopathy has been reported in these patients.

We describe two individuals with a homozygous stop mutation in the SPTBN4 gene. The patients presented with muscular hypotonia, muscular atrophy and severe psychomotor developmental arrest. Deafness is present in one of them.

Since patient I presented with cardiomyopathy, optic atrophy and MRI pathologies we were interested to determine if mitochondrial dysfunction was present in the muscle which was supported by the biochemical examination.

Here, we present the first case of a severe form of ßIV-spectrin deficiency with cardiac involvement and mitochondrial dysfunction.

## Materials and Methods

### Whole-Exome Sequencing

Whole exome-sequencing in the DNA of patient I and II was performed as described by Park et al. (16). Selection parameters were used as described in Reunert et al. (17). Only variants that were found in both patients were further considered.

The mutations were confirmed by Sanger sequencing, patients are homozygous, the mother heterozygous, as well as the healthy sister who is also heterozygous.

### Electron Microscopy of the Sural Nerve

For electron microscopy tissue was fixed in 2.5% buffered glutaraldehyde. Samples were transferred to Sorensen's phosphate buffer (pH7.2). The tissue was dehydrated through ascending alcohols before embedding in epon. Epon blocks were trimmed and 1 μm sections were cut, stained with toluidine blue for epoxy semi-thin sections and examined by light microscopy. Regions of interest were chosen by light microscopy. Ultrathin sections were cut with a diamond blade onto copper grids using an ultramicrotome. Copper grid-mounted ultrathin sections were stained with uranyl acetate (77870, Serva) and Reynold's lead citrate and examined by transmission electron microscopy.

### Biochemical Examination of the Muscle Tissue

Biochemical examination of the muscle tissue was performed as described by Gödiker et al. (18).

## Case Description

### Clinical Presentation of Patient I

The male patient was born to healthy consanguineous Syrian parents with a normal birth weight after an uneventful pregnancy.

The patient has an older sister who is healthy and heterozygous carrier of the SPTBN4 mutation and a younger sister who was also diagnosed with ßIV-spectrin deficiency and described as patient II below. According to the patients' mother no other family member is known to present with a similar phenotype or symptoms.

After birth the patient presented with general muscular hypotonia and areflexia, as well as global muscular atrophy. Twelve months later, the patient had not reached any milestone and did not show any muscular movements (Table 1).

**Table 1:** Comparison of the major symptoms of patient I and patient II.

**Symptoms**	**Patient I Male, 17 years**	**Patient II Female, died at the age of 14 months**
**Neurological symptoms**		
• General muscular hypotonia	+	+
• Muscular atrophy	+	+
• Areflexia	+	+
• Sluggish sucking reflex	+	+
• Severe psychomotor developmental arrest	+	+
• Absent language development	+	+
• Brain MRI abnormalities	+	+
Visual and auditory symptoms		
• Optical nerve atrophy	+	
• Deafness	+	
**Respiratory symptoms**		
• Respiratory failure	+	+
• Recurrent pneumoniae	+	+
**Cardiological symptoms**		
• Hypertrophic cardiomyopathy of the non-obstructive type	+	
**Gastroenterological symptoms**		
• Dysphagia induced malnutrition	+	+
• Reflux disease	+	+
• Gastrostomy tube/nasogastric tube	+	+
**Symptoms of the musculoskeletal system**		
• Inactivity osteoporosis and bone fractures	+	
• Scoliosis/hyperlordosis/hypokyphosis	+	

To identify the cause of his severe psychomotor delay, a muscle biopsy was taken at the age of 5 month which showed unsuspicious skeletal muscle without indication of any myopathic, neurogenic or inflammatory changes. Muscular biopsy from vastus muscle at the age of 2 years showed slightly widened fiber caliber spectrum which has been classified as mild hypertrophy of the muscle fibers.

**Figure 1:**
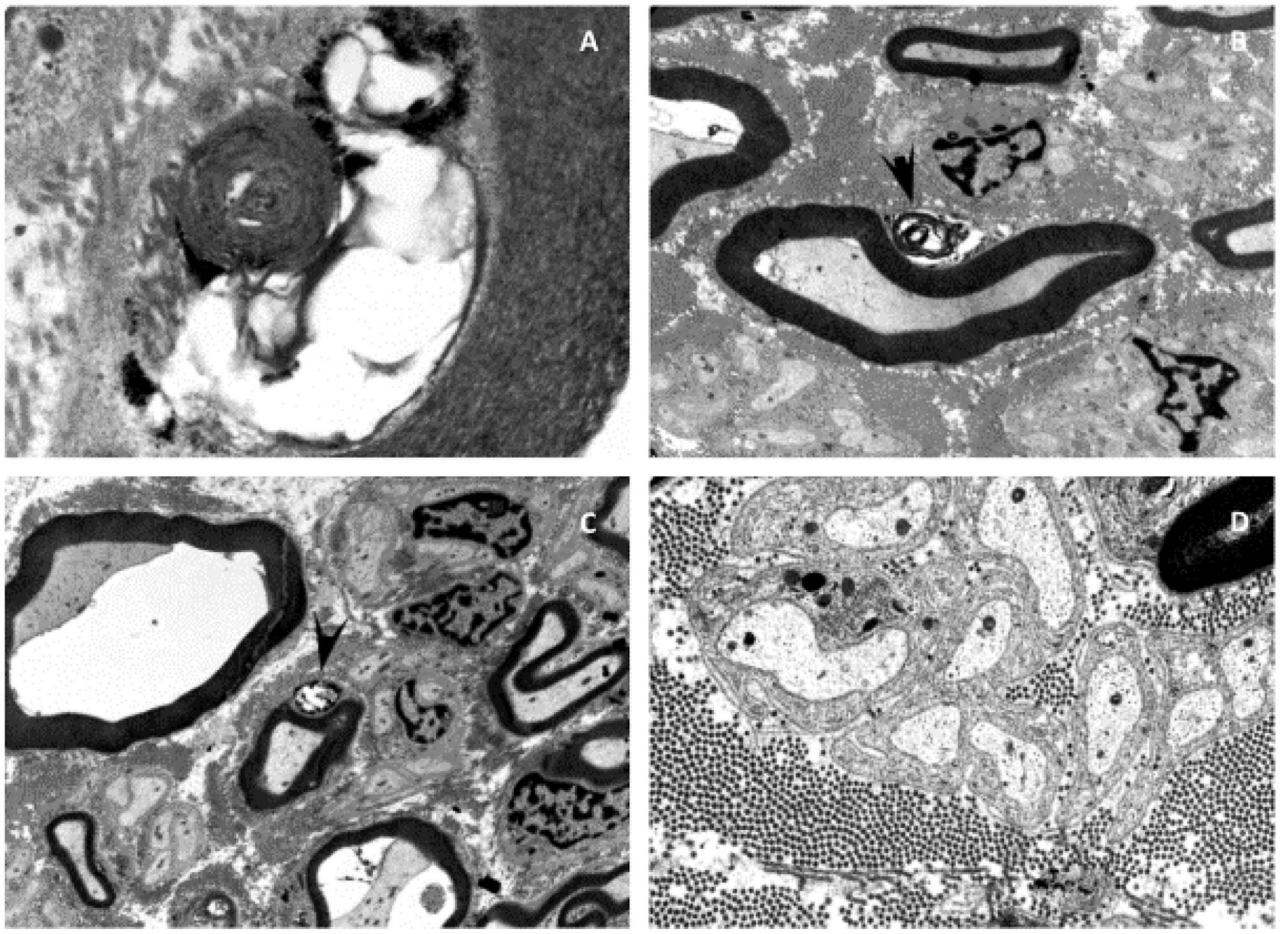
Electron microscopy of the sural nerve of patient I. Electron microscopy showed neurodegenerative processes and inclusions in the Schwann cells of myelinated axons of the sural nerve **(A–D)**. Some of these inclusions can be identified as myelin-like figures with concentric lamellar material and periodicity of about 10 nm **(A)**. The inclusions are often mixed with glycogen-like granules **(C)**. Membranous vacuolized and optically empty bodies, as well as isolated irregularly contoured electron-dense and sharply delimitable “tufa”-like inclusions **(B,C)**. These inclusions lead to the suspicion of a lysosomal storage disease.

Electron microscopic examinations of nerve biopsies at the age of 2 years showed membrane inclusions in the swan cells of myelinated axons, as well as demyelinating changes, raising the suspicion of a lysosomal storage disease (Figure 1).

A mitochondrial association was considered owing to the symptom complex of severe psychomotor delay, cardiomyopathy and visual involvement. Biochemical examination of the muscle biopsy revealed a disturbed mitochondrial energy generating system, with reduced activities of complexes II, III, II+III and, in particular, of complex I (Table 2).

**Table 2:** Results of the mitochondrial enzyme diagnostics.

	**Activity**	**Control range**	**Unit**
**Substrate oxidation rates**			
1. [1-^14^C] pyruvate+ malate	0.74	3.61–7.48	nmolCO2/h.mUCS
2. [1-^14^C] pyruvate + carnitine	1.18	2.84–8.24	nmolCO2/h.mUCS
3. [U-^14^C] malate + pyruvate + malonate	0.80	4.68–9.62	nmolCO2/h.mUCS
4. [U-^14^C] malate + acetylcarnitine + malonate	1.15	3.43–9.62	nmolCO2/h.mUCS
5. [U-^14^C] malate + acetylcarnitine + arsenite	0.93	2.05–3.85	nmolCO2/h.mUCS
6. [1,4-^14^C] succinate + acetlycarnitine	0.70	2.54–6.39	nmolCO2/h.mUCS
**ATP metabolism**			
ATP + CrP production pyruvate	7.6	42.1–81.2	nmol/h.mUCS
**Enzyme activities**			
Complex I	20	70–251	mU/UCS
Complex II	291	335–749	mU/UCS
Complex III	1,523	2,200–6,610	mU/UCS
Complex II + III (Succ.: cyt.c oxidoreductase; SCC)	222	300–970	mU/UCS
Complex IV	825	810–3,120	mU/UCS
Complex V (ATPase)	345	169–482	mU/UCOX
Citrate synthase	37	37.4–162	mU/mg

*Oxidation rates for ^14^C-labeled substrates were calculated as nmol ^14^CO_2_/(h · mU CS). The ATP production rate was calculated as nmol (ATP + CrP)/(h mU CS). Complex I activity was expressed as mU/U complex II, mU/U complex IV, or U/g protein, in which 1 U complex I activity equals 1 μmol DCIP reduced per min. The activity of complex I is significantly reduced in patient I's muscle. Complex II, III, II + III activity is also reduced*.

Laboratory parameters like lactate and CK in serum were always within the normal range.

At the age of 16 months, electroencephalogram (EEG) recordings showed predominant delta and theta activity but no seizure activity.

**Figure 2:**
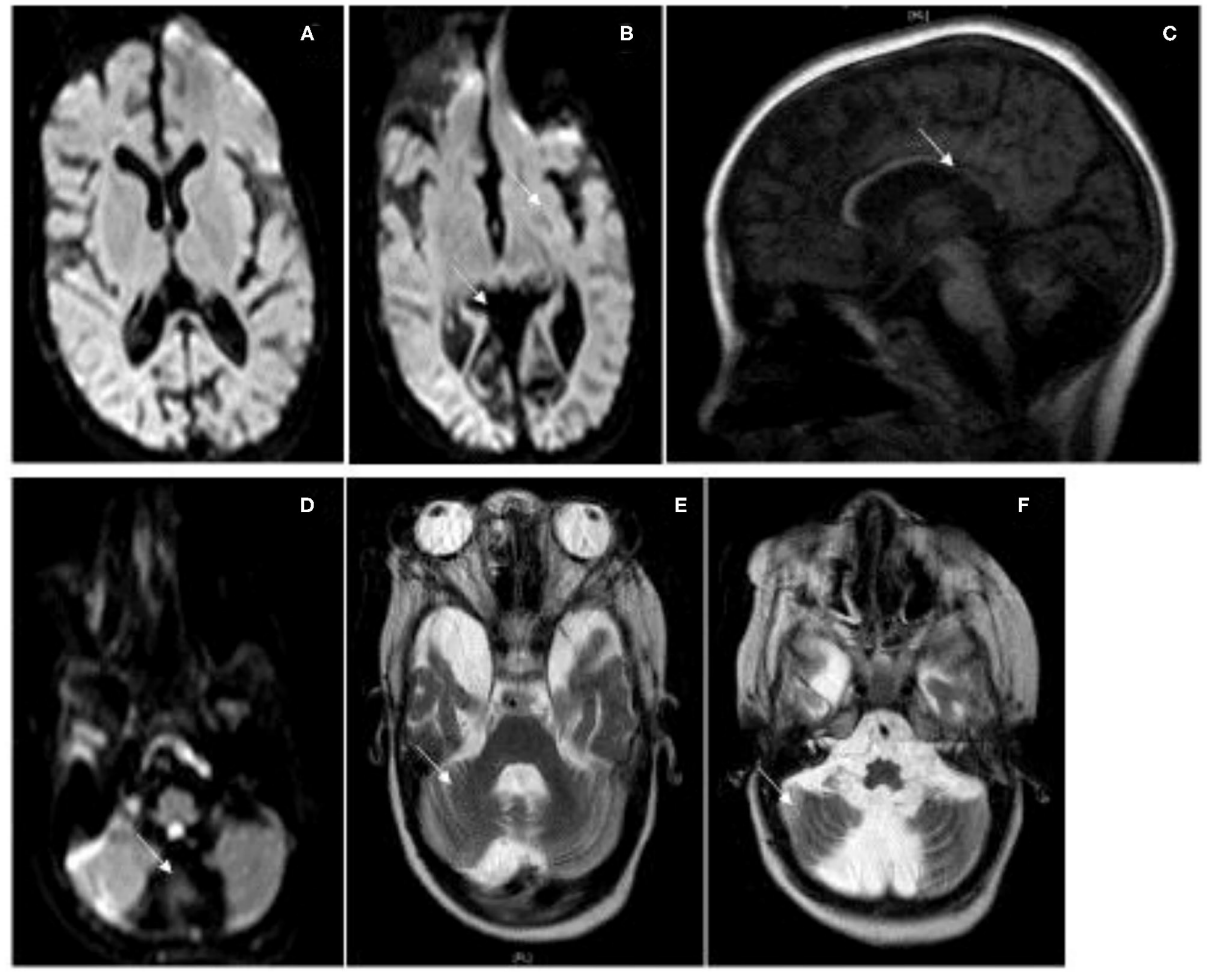
MRI Images. Cranial MRI of patient I at the age of 10 years. **(A,B)** Flair, **(C)** T1, **(D)** Flair, **(E,F)** T2. MRI shows severe atrophy of the cerebral cortex especially of the occipital cortex and a severe atrophy of the cerebellar hemispheres with a loss of the cerebellar vermis, a thin corpus callosum, and optical atrophy.

Cranial magnetic resonance imaging (MRI) at the age of 18 months showed a global cerebral atrophy, while cranial MRI at the age of 10 years showed signs of progressive, focal brain atrophy and a continuing but not age-appropriate myelination. In particular, the MRI revealed an atrophy of the occipital cortex accentuated in the occipitotemporal medial gyrus, an almost entire loss of the cerebellar vermis and a significant loss of substance of the cerebellar hemispheres and a temporopolar dilation of the subarachnoid cavity and corpus callosum hypoplasia (Figure 2).

Electroneurography (ENG) of the ulnar and the tibial posterior nerve was performed at the age of two and a half years and was difficult to evaluate. However, ulnar nerve showed a nerve conduction time of 6,08 m/s (norm: 59,8), slight muscular reaction in both muscles was observed up to 30 mA. ENG showed a deceleration of nerval conduction.

The patient shows severe optic atrophy causing blindness.

Additionally, the patient suffers from deafness.

He suffers from chronic respiratory insufficiency due to failure of the ventilatory motor function with a high production of mucus and an insufficient cough reflex. Tracheostomy and a home ventilation device were necessary already at the age of 1 year. Pulmonary parenchyma infections with fever have been occurring in regular intervals since birth.

Echocardiography at the age of 1 year revealed a non-obstructive hypertrophic cardiomyopathy.

Due to muscular hypotonia and a sluggish sucking reflex, he had to be fed by nasogastric tube after birth. At the age of 1 year he showed a malnutritional status caused by dysphagia, which indicated the application of a percutaneous endoscopic gastrostomy tube.

In the sequel of a cardia insufficiency the patient developed a duodeno-gastro-esophageal reflux disease at the age of 8 years. Furthermore, the patient had a corpus and a fundus gastritis.

Orchidectomy had to be performed due to haemorrhagic necrosis of his inguinal testis at the age of 12 years.

Due to an inactivity osteoporosis and after two fractures of the femur, patient I receives a bisphosphonate therapy. He developed a thoracolumbar scoliosis, thoracic hypokyphosis and lumbar hyperlordosis (Table 1).

**Figure 3:**
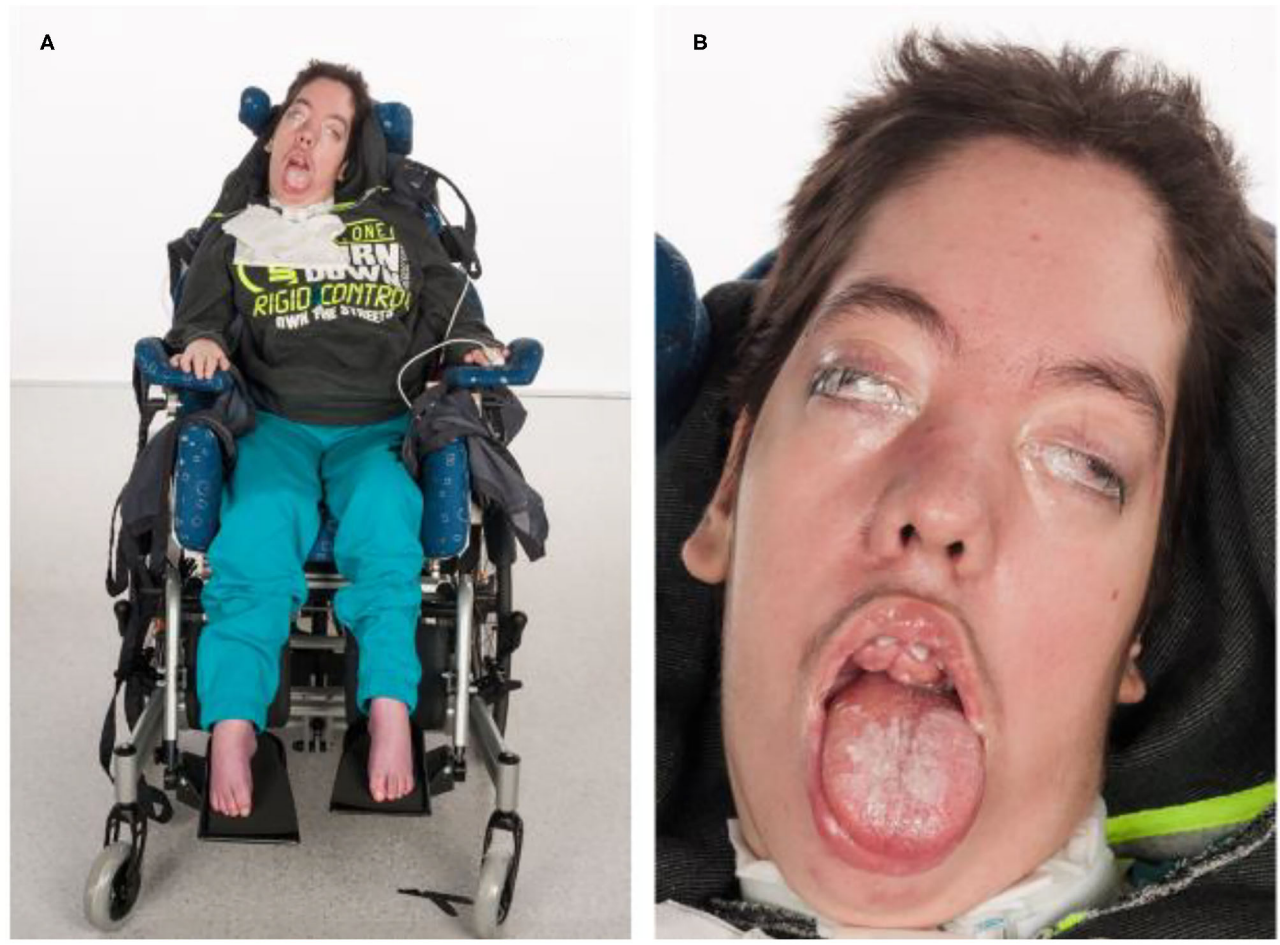
Phenotype of patient I at the age of 15 years. **(A)** Severe global muscular hypotonia, scoliosis, talipes equinus. **(B)** Facial dysmorphias: Long drawn face with a high forehead, hypertelorism and exophtalmus, opened mouth, macroglossia, gingiva hyperplasia with teeth anomalies, small philtrum, and prominent eyebrows.

Currently, at the age of 17, the patient shows severe psychomotor developmental arrest, myopathic facies, a long-drawn face with a high forehead and a palate column. He has a gingiva hyperplasia with a malformation of his teeth, growing out of his palate. The patient's mouth is always open and he has a macroglossia in relation to his oral cavity. Moreover, he has a vertical and horizontal gaze palsy and hypertelorism (Figure 3). Since he is unable to communicate verbal and non-verbal by eye movements or mimics, the only reactions to his environment that can be detected are the changing of his heart rate or respiratory rate. Unspecific movements, for example, of his tongue or his extremities can be observed seldom. The patient is in a wheelchair and unable to perform any basic actions.

### Clinical Presentation of Patient II

The younger sister of patient I was born with a birth weight of 3,920 g after 40 weeks of an uneventful pregnancy.

She presented as a floppy infant, with distinct muscular hypotonia and missing sucking reflex. Clinical examination showed sparse symmetrical spontaneous motoric function.

MRI showed an external more than internal hydrocephalus leading to global cerebral atrophy, in particular of the periventricular space and the corpus callosum. The myelination was age-appropriate.

Clinically she showed microcephaly.

Like her older brother, she was suspected to have a lysosomal storage disease.

Patient II was diagnosed with chronic global respiratory insufficiency since she suffered from recurrent pulmonary parenchyma infections with poor mobilization of secretions.

Because of her missing sucking reflex, feeding was difficult and she developed a severe malnutrition necessitating gastric tube feeding. Furthermore, the patient developed a duodeno-gastro-esophageal reflux disease (Table 1).

Patient II died at the age of 14 months due to a bronchopulmonary infection in the course of which she developed a rapidly decreasing oxygen saturation and an increasing bradycardia.


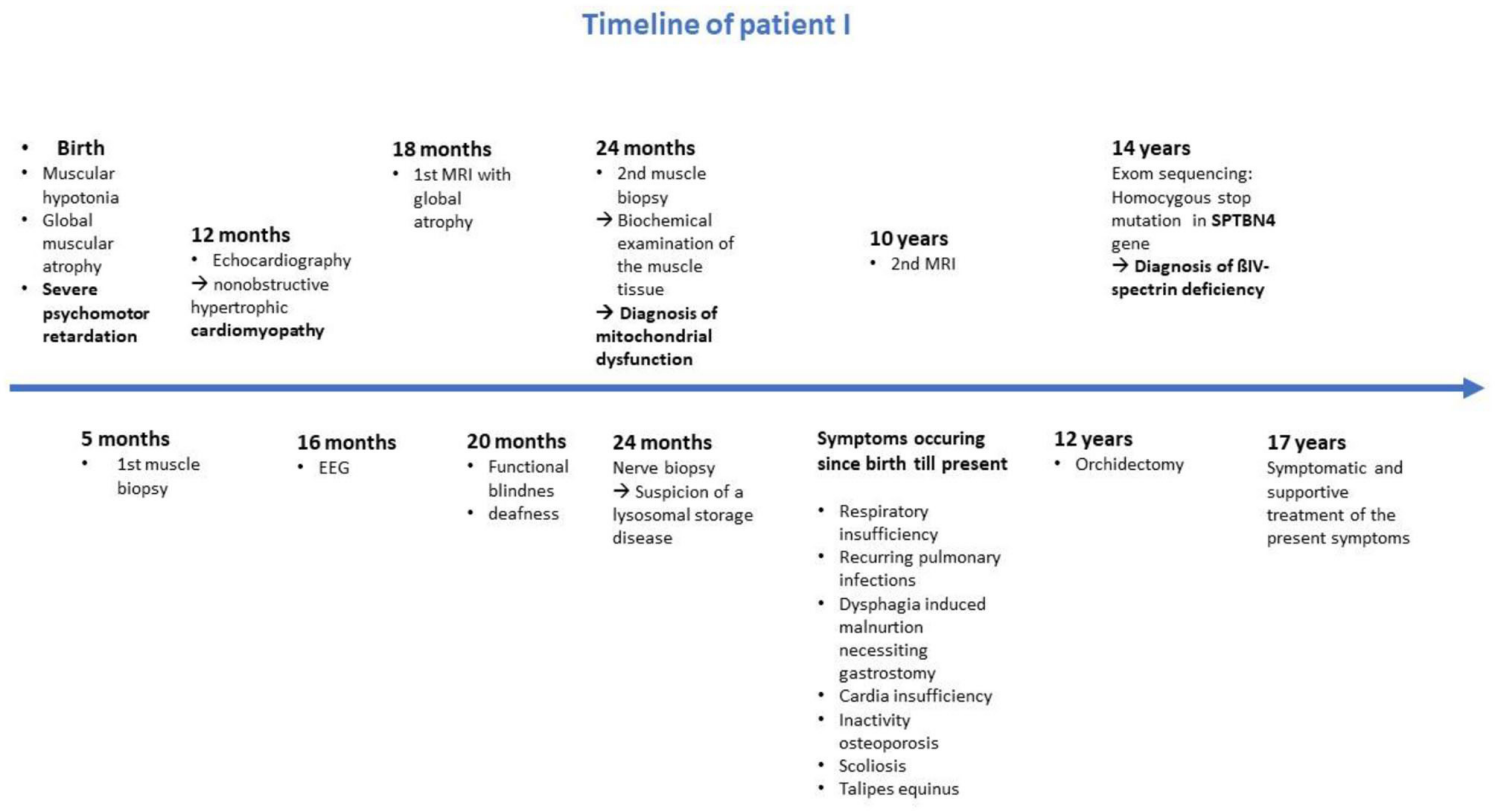


## Diagnostic

### Exome Sequencing

Whole exome sequencing of patients I and II revealed the homozygous stop mutation c.6016C>T (p.R2006^*^protein with 2564 aa, Mut. Ex 28 of 36) in the SPTBN4 gene in both patients. Homozygous carrier status was confirmed by Sanger sequencing in both patients.

Heterozygous carrier status of the mother and the healthy sister was confirmed by Sanger sequencing as well.

DNA of the father has been unavailable.

Other genetic variants that were found in both patients included variants in the genes BNIPL, FBLN7, GABPB2 (homozygous) and PLEC (heterozygous). After using the above mentioned analyses parameter and additional prediction programs (PROVEAN, SIFT, Polyphen2), these mutations were excluded as they were either predicted to be benign by the majority of prediction programs or due to publication data that revealed gene functions that were not indicative for the disease of patient I and II.

### Biochemical Examination of Muscle Tissue

Biochemical examination of muscle tissue revealed a disturbed energy generating system.

A severely reduced substrate oxidation and ATP + CrP production rates were detected in the muscle tissue. At the enzymatic level, a clearly reduced activity of complex I was observed. Also complex II, III, and II+III showed reduced activities, although not as severe as complex I. The activities of complex IV and V were normal. Activity of the reference enzyme citrate synthase was around the lowest control value. There was not enough material left to measure PDHc.

## Discussion and Conclusions

ßIV-spectrin deficiency is still a rarely known disease. It is caused by mutations in the SPTBN4 gene. Since ßIV-spectrin, actin and ankyrin G play a major role in the preservation of the integrity of the AIS and the nodes of Ranvier, lack of or deficiency of ßIV-spectrin leads to a disorganization and misclustering of the neuronal cytoskeleton and destabilization of neuronal cytoskeletons subsequently (13). As ion channels like KCNQ potassium-channels and NaV sodium channels being organized in the cytoskeleton membrane by the interaction of ßIV-spectrin and ankyrin G, SPTBN4 mutation induces their destabilization and misfunctioning (19). Thus, the generation of the action potential is disturbed (5). This mechanism leads to a neuropathy followed by a secondary myopathy.

While Knierim et al. described the mutation in the SPTBN4 gene to cause a congenital myopathy with absence of fiber type I muscle, Wang et al. described a neuropathy and a secondary myopathy, since he observed motor axonal neuropathy and reduced Na+- and K+–channels in neurons (14, 15). The pathogenesis remains not completely understood. However, our results indicate a neuropathy with a secondary myopathy, since muscle biopsy after birth did not reveal any pathological alterations and electron microscopy of a peripheral nerve showed neurodegenerative process with undefined inclusions of the Schwann cells. The second biopsy at the age of 2 years showed mild hypertrophy of the muscle tissue, which was not considered as typical for a primary myopathy, indicating a pathological process in the muscle since the first biopsy had been taken.

All seven patients with ßIV-spectrin-deficiency already published in literature, presented a phenotype with general muscular hypotonia, muscular atrophy, facial weakness and severe psychomotor delay, feeding difficulties inducing artificial feeding, as well as central deafness (14, 15). The six patients identified by Wang et al. also suffered from blind sight, seizures and respiratory difficulties (15).

In accordance with the described clinical phenotypes our patients present congruent symptoms, like congenital muscular hypotonia and atrophy, severe psychomotor developmental arrest, respiratory insufficiency, dysphagia induced malnutrition and in case of the first patient deafness. In contrast to these already published patients, patient I additionally shows MRI changings, optical nerve atrophy and cardiomyopathy. Biochemical examination revealed a deficiency of complex II, III, II and III, and in particular, of complex I of the respiratory chain (Table 2). Whole exome sequencing did not reveal any other genes being possibly responsible for the mitochondrial dysfunction.

Recently, ßIV-spectrin was found to play an important role in the heart, where it is located in the myocyte intercalated discs and organizes ion channels like Na+- or K+-channels e.g., TREK-1 being important for the myocardial action potential (9).

A mouse model with ßIV-spectrin mutant mice has shown impaired cardiac function through different mechanisms, involving ßIV-spectrin (10, 11). Myocytes of SPTBN4 mutant mice have shown highly eccentric myocyte growth (11). ßIV-spectrin was found to bind the transcription factor STAT3, a transcriptional regulator influencing cell responses like hypertrophy, proliferation and inflammation (11). Patel et al. has shown that loss of ßIV-spectrin leads to increased fibrosis through activation of STAT3 and thus to cardiac dysfunction (11). These results suggest that the hypertrophic cardiomyopathy of patient I could be the consequence of ßIV-spectrin deficiency. Nevertheless, it remains elusive whether the cardiomyopathy is caused by the SPTBN4 mutation or occurs in the sequel of another pathological process.

Moreover, patient I also shows specific facial features with a long drawn face, a high forehead, hypertelorism, gingiva hyperplasia, teeth anomalies and opened mouth, macroglossia, small philtrum and prominent eyebrows. Facial features could also be caused by other congenital diseases, however WES did not reveal another gene possibly responsible. The myopathic facies does not correlate specifically to any other known congenital diseases.

Prior to the exome analysis, a lysosomal storage disease had been suspected due to inclusions in the Schwann cells of myelinated axons seen in the electron microscopy (Figure 1). Saifetiarova et al. has detected similar inclusions and deformations of myelinated axons due to mutations in the SPTBN4 gene in mutant mice resulting in demyelination and neurodegeneration of central and peripheral axons (12). These findings correspond to the general demyelination seen in the MRI of index patient I. ßIV-spectrin seems to play a major role in the preservation of the integrity and myelination process of axons.

The combination of genetic, biochemical examination and the comparison of clinical symptoms led to the final diagnosis of a ßIV-spectrin deficiency, underlining the importance of comprehensive diagnostics in patients presenting with a various range of different symptoms not matching to any known disease or etiology.

Due to missing material and ethical reasons further diagnostic could not have been performed. For future patients it would be interesting to make further investigations at an early state of the disease. For example, immunohistochemical examination to visualize ßIV-spectrin in the muscle tissue and the nerve biopsy. Furthermore, electron microscopy of the muscle and nerve in order to see possible morphological alterations of the mitochondria. To further study the role of ßIV-spectrin in the heart endomyocardial biopsies would help to determine the origin of the hypertrophic cardiomyopathy.

However, in context of consanguinity it can not be excluded, that other not detected mutations, were present and relevant for mitochondrial function. Variants within non-analyzed regions of the analyzed genes, e.g., untranslated regions, introns, promoters or enhancers, some types of repeat expansions as well as deletions or duplications of several exons or whole genes can not be excluded and might be also relevant for mitochondrial function. The same is true for genomic regions that are not accessible to exome analysis due to technical limitations (GC-content, homologous pseudogenes etc.).

But since whole-exome-sequencing of patient I did not reveal a gene causing the mitochondrial dysfunction the question arises how ßIV-spectrin-deficiency and mitochondrial dysfunction could be related.

Mitochondrial dysfunction has been identified in a various range of neurodegenerative and neuromuscular diseases, for instance, Alzheimer's disease, Huntington's disease, muscular dystrophies, Amyotrophic lateral sclerosis, Parkinson's disease, traumatic brain injury or stroke (20).

One model to explain mitochondrial dysfunction in these diseases could be due to missing innervation of the muscle, muscle replacement by fat cells or fibroblasts and hence a decreased activity of the mitochondria. Patients with neuropathies showed a significantly lower activity of complexes I-III (21).

**Figure 4:**
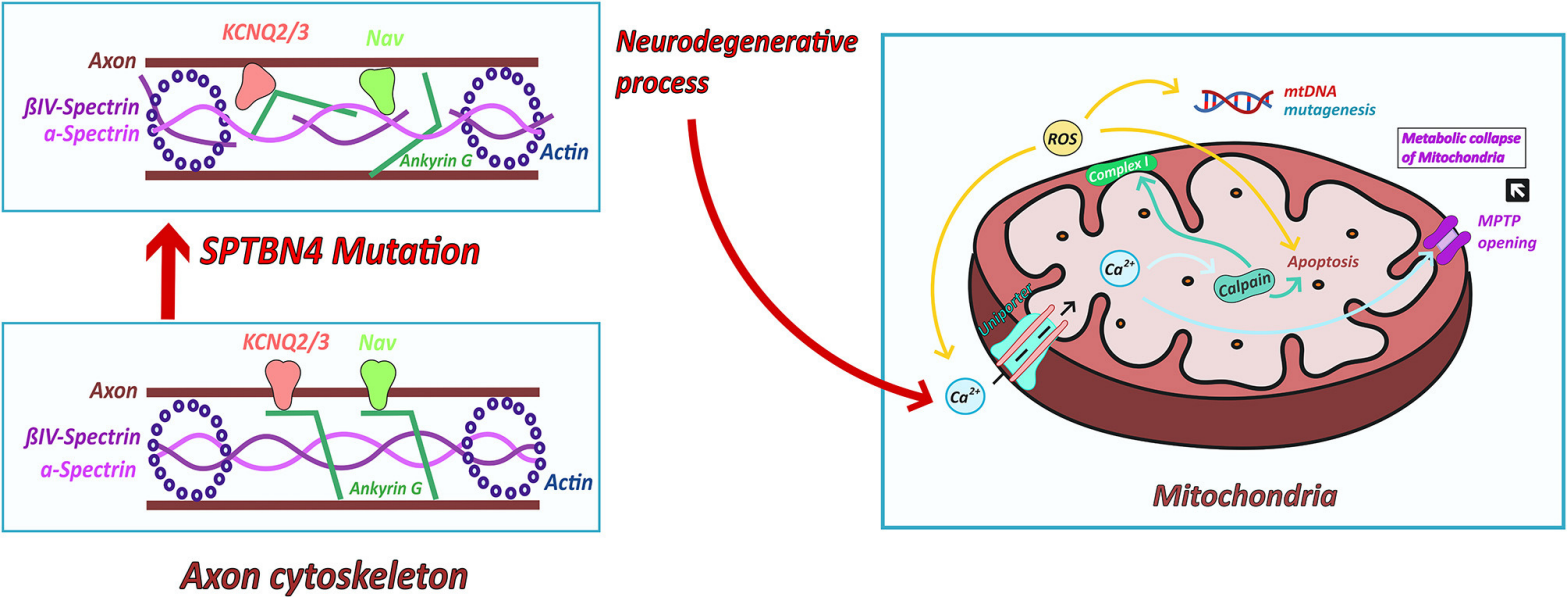
Possible links between ßIV-spectrin deficiency and mitochondrial dysfunction. ßIV-spectrin deficiency leads to a destabilization of the cytoskeleton and thus to misclustering of ion channels, like NaV sodium and KCNQ potassium channels. The resulting neurodegenerative process could cause an altered calcium homeostasis (23). Increased calcium concentration enter the mitochondria via ruthenium red sensitive Ca^2+^ uniporter inducing the activation of calpain. Calpain induces impaired respiratory chain activity (23) and cell death (24, 25). Furthermore, increased calcium influx leads to opening of MPTP and thus swollen mitochondria and metabolic collapse of the mitochondria. Complex I produces ROS which leads to increasing calcium concentration and cell death. ROS are produced in complex I (22). Increasing oxidative stress leads to cell stress and to liberation of calcium from the ER. ROS could then induce mtDNA mutagenesis and neuronal cell death (20).

Another model which has been studied in multiple neurodegenerative and neuromuscular diseases is an increased production of ROS (Reactive oxygen species) produced in complex I and III (22). Increasing oxidative stress leads to cell stress and to liberation of calcium from the ER (endoplasmatic reticulum). ROS could then induce neuronal cell death and mtDNA mutagenesis (20) (Figure 4). The opening of the mitochondrial permeability transition pore (MPTP) activated by calcium overload results in osmotic swelling, uncoupling of the electron transport and metabolic collapse of the mitochondria (26) (Figure 4).

Moreover, altered calcium homeostasis has been reported to induce calpain activation leading to mitochondrial disturbance and cell death (23). Arrington et al. (23) has identified calpain-10 to cause mitochondrial dysfunction through the cleavage of complex I subunit's (24). Furthermore, different forms of calpains contribute to apoptotic cell death pathways (24, 25) (Figure 4).

Another model to explain mitochondrial dysfunction in neurodegenerative diseases, is the disturbance of mitochondrial fission and fusion processes (27, 28). Ordonez et al. has reported the impairment of the spectrin cytoskeleton by α-synuclein leading to mitochondrial dysfunction (29).

Given the common secondary mitochondrial involvement in neurodegenerative diseases, our hypothesis of a possible connection between ßIV-spectrin deficiency and mitochondrial dysfunction will have to be to be investigated experimentally in further studies.

In conclusion, we here presented the first patient with a severe form of ßIV-spectrin-deficiency including the classical symptoms of ßIV-spectrin deficiency, but furthermore mitochondrial dysfunction and hypertrophic cardiomyopathy. Our results emphasize that ßIV-spectrin deficiency is still a sparsely known disease with a wide range of different symptoms being difficult to diagnose. Further patients need to be described or tested to investigate whether cardiomyopathy and mitochondrial dysfunction are associated with ßIV-spectrin deficiency.

In view of therapeutical targets these findings could open new options for future patients.

### Patient Perspective

Due to the severe psychomotor developmental arrest of the here presented patient I, it is difficult to evaluate his perspective on the illness he is suffering from. According to the patient's mother and people who accompanied the patient since his infancy, patient I is able to register and feel if the person around him is someone he is familiar with. In stressful situations in the hospital, for example, the heart rate as well as the blood pressure increase. In rare situations uncontrolled movements, for instance, from the tongue can be detected. The severe course of patient I's disease emphasizes the importance of an early diagnosis and detection of rare illnesses.

## Data Availability Statement

The original contributions presented in the study are included in the article/supplementary material, further inquiries can be directed to the corresponding author/s.

## Ethics Statement

Written informed consent was obtained from the individual(s), and minor(s)' legal guardian/next of kin, for the publication of any potentially identifiable images or data included in this article.

## Author Contributions

TM contributed mainly to the clinical examination of the patient, diagnostic process and coordinated and supervised data collection, and critically reviewed the manuscript for important intellectual content. MF has performed clinical examinations in the childhood of the patient and was mainly involved in diagnostic processes. AJ has performed electron microscopy and analyse of the electron microscopy images. SR was involved in the analysis of the whole exome sequencing. AS has performed Sanger sequencing. RR and LvdH have performed mitochondrial examination and interpretation and critically reviewed the manuscript. CE has mainly contributed to the clinical part and the MRI imaging as well as the progress of the discussion part and and critically reviewed the manuscript for important intellectual content. JR has contributed mainly to the genetical part and interpretation of the whole exome sequencing and critically reviewed the manuscript. AB has written the manuscript, conceptualized and designed the case report, collected, analyzed and interpreted the data, drafted the initial manuscript, developed the ideas of possible connections between ßIV-spectrin defiency and mitochondrial dysfunction, and reviewed and revised the manuscript. All authors approved the final manuscript as submitted and agree to be accountable for all aspects of the work.

## Conflict of Interest

The authors declare that the research was conducted in the absence of any commercial or financial relationships that could be construed as a potential conflict of interest.

## Abbreviations

AIS: Axon initial segment
STAT3: Transcription 3
EEG: Electroencephalogram
MRI: Magnetic resonance imaging
ENG: Electroneurography
DNA: Deoxyribonucleic acid
ATP: Adenosine triphosphate
CrP: C-reactive protein
PDHc: Pyruvate dehydrogenase complex
ROS: Reactive oxygen species
MPTP: Mitochondrial permeability transition pore.
